# Hepatitis B and C in Pakistan: A community survey

**DOI:** 10.7759/cureus.5992

**Published:** 2019-10-24

**Authors:** Nismat Javed, Muhammad Hassan Nadeem, Haider Ghazanfar

**Affiliations:** 1 Internal Medicine, Shifa College of Medicine, Shifa Tameer-E-Millat University, Islamabad, PAK; 2 Public Health, Shifa College of Medicine, Shifa Tameer-E-Millat University, Islamabad, PAK

**Keywords:** pakistan, hbv, hcv, awareness

## Abstract

Objective

To assess the efficacy of awareness campaign aimed at Hepatitis B and C in a rural community in Rawalpindi, Pakistan.

Methods

This is a cross-sectional study conducted on patients from a primary health care facility in Rawalpindi. The duration of the study was three months (April 2019 to June 2019). Patients who were registered at the facility were included after consent had been obtained. The data was collected through a self-constructed questionnaire. The data obtained was analyzed on IBM's statistical package for the social sciences (SPSS) version 21 (IBM, Armonk, NY).

Results

Out of 35 participants, 16 (46%) were males and 19 (54%) were females. The mean age of the participants was 43.82 ± 19.56 years. The mean number of people in the house was 7.54 ± 3.88. Twenty nine (84%) participants were married while six (16%) participants were not. A majority of the participants had good prior knowledge about Hepatitis B and C. However, a few confusions remained about the mode of transmission, the vector for transmission if any and vaccination protocols. The campaign proved to reinforce many concepts and clear potential confusions of the participants.

Conclusions

This attempt at improving awareness proved to be fruitful. There is a dire need to ensure that multiple activities are organized so that the burden of the disease may be reduced. There will be a strong network of communication for flow of information if the activities occur regularly and in a focused manner.

## Introduction

Hepatitis B virus has infected over 240 million people worldwide with about half of the cases progressing to a chronic phase [1]. Similar statistics are available for Hepatitis C [2]. A large percentage of infectious disease burden in developing countries can be attributed to these two viruses [3]. Each of the viruses has a peculiar characteristic responsible for chronicity.

Hepatitis B virus (HBV) has a complex mechanism of liver injury in terms of liver damage and viral control and these factors are primarily responsible for the delayed diagnosis and subsequent progression to chronic illness. Hepatitis C virus (HCV) on the other hand has a multitude of genotypes [4]. The most prevalent and virulent genotype is HCV genotype 1 followed by genotype 3 [5]. Therefore, in both these diseases, the question of creating a vaccine and subsequently, its thorough implementation is still unanswered [6-7].

The route of transmission of both these viruses are more or less similar and therefore, awareness campaigns and public health awareness measures deal with both of the infections as a combined entity [8]. The disease presentation can be very diverse. Common symptoms are characterized by right upper-quadrant abdominal pain, fatigue, jaundice and arthralgia [9]. However an acute episode superimposed on chronic illness with non-specific symptoms, for example arthralgia or fatigue, may also be seen [10-12].

A common misconception centers on the myth that these viruses are transmitted by insect vectors, such as mosquitoes. A few cases have been reported of drug abusers being infected with diseases due to insect vectors but hepatitis continues to be the exception.

The objective of the study was to assess the efficacy of a public health awareness activity centered on a rural community at a Primary Health Care facility in Rawalpindi, Pakistan.

## Materials and methods

We conducted a cross-sectional study on a small community who were enrolled at a primary health care facility from April 2019 to June 2019. Patients who were willing to participate in the study were included. Patients who were not present on the days of the study were not included in the study.

The data collection was divided into two parts. The first part of data collection was the administration of a self-constructed questionnaire prior to providing the patients information about Hepatitis B and C. This questionnaire was developed with the help of a prior questionnaire and advice from public health education experts [13]. There were four sections in the questionnaire. The first section contained questions pertaining to the demographic information of the participants. The second section contained questions assessing the knowledge of participants regarding transmission of Hepatitis B and C. The third section focused on symptoms of the disease while the fourth section contained questions related to prevention. Table 1 shows the number of questions contained in each section.

**Table 1:** Number of questions in each section

Section	Number of questions
Demographic details	9
Transmission of Hepatitis B and C	10
Symptoms of disease	2
Prevention of disease	3

The responses, apart from demographic details, were all scored. Each correct answer scored one point and each wrong answer scored zero point. The "Do not know" response was considered a wrong answer and scored zero point.

The questionnaire was distributed to all participants. All the participants were given 20 minutes to complete the questionnaire. After the data collection, all the students were given a seminar on Hepatitis B and C. A question and answer session was held after the seminar to address any queries regarding Hepatitis B and C. The same questionnaire was administered at the end of the study period for post-awareness responses.

The data obtained was analyzed on IBM's statistical package for the social sciences (SPSS) version 21 (IBM, Armonk, NY). Descriptive statistics were used to analyze and describe the data. Frequencies and percentages were calculated for qualitative variables like gender. Mean and standard deviation (SD) were calculated for quantitative variables like age.

## Results

Out of 35 participants, 16 (46%) were males and 19 (54%) were females. The mean age of the participants was 43.82±19.56 years. The mean monthly household income was 26114 ± 12538 rupees. The mean number of people in the house was 7.54 ± 3.88. Twenty nine (84%) participants were married while six (16%) participants were not. The pre-awareness and post-awareness scores were then calculated. Table 2 tabulates the responses related to transmission of disease. A paired samples t-test was used to determine any significant differences. P-value less than 0.05 was considered to be significant.

**Table 2:** Transmission of Hepatitis B and C S.D.= Standard Deviation P-value less than 0.05 was considered significant

STATEMENT	PRE-AWARENESS SCORE (Mean±S.D.)	POST-AWARENESS SCORE (Mean±S.D.)	p-value
Hepatitis B and C are spread by shaking hands or speaking to a patient	0.80±0.49	0.71±0.46	<0.05
Hepatitis B and C are transmitted by a pregnant mother to her fetus	0.74±0.44	0.77±0.43	>0.05
Are the diseases spread by sexual activities?	0.69±0.47	0.83±0.38	>0.05
Does the disease spread by interacting with close contacts of the same household?	0.66±0.48	0.40±0.22	<0.05
Can transmission of Hepatitis B be reduced by vaccination?	0.86±0.36	0.86±0.36	>0.05
Are the diseases transmitted by consumption of food contaminated with the viruses?	0.78±0.35	0.39±0.19	>0.05
Are the diseases transmitted by mosquito bites?	0.62±0.30	0.31±0.11	>0.05
Hepatitis B and C are spread by many people using the same syringe	0.91±0.35	0.94±0.23	>0.05
Carriers of the disease can marry each other	0.31±0.11	0.54±0.35	<0.05
Diseases are spread by using the syringe of an infected patient	0.86±0.36	0.94±0.24	>0.05

Table 3 tabulates the responses related to symptoms of disease. Paired samples t-test did not show significant differences in this case.

**Table 3:** Symptoms of Hepatitis B and C S.D.= Standard Deviation

STATEMENT	PRE-AWARENESS SCORE (Mean±S.D.)	POST-AWARENESS SCORE (Mean±S.D.)
Are fever, malaise, nausea and jaundice symptoms of both diseases?	0.89±0.32	0.99±0.17
Does liver cirrhosis signify chronic phase that may lead to carcinoma?	0.83±0.38	0.91±0.28

Table 4 shows the scores for prevention of disease. Paired samples t-test did not show significant differences in this case.

**Table 4:** Prevention of Hepatitis B and C S.D.=Standard Deviation

STATEMENT	PRE-AWARENESS SCORE (Mean±S.D.)	POST-AWARENESS SCORE (Mean±S.D.)
Hepatitis B vaccine is one of the effective ways for preventing the disease	0.65±0.25	0.80±0.19
Does Hepatitis B vaccination protect against Hepatitis C?	0.34±0.10	0.20±0.09
Hepatitis B vaccination is included in Extended Program for Immunization	0.54±0.21	0.66±0.40

## Discussion

This is the first study in which efficacy of an awareness activity has been investigated as opposed to prior knowledge of the people or the general prevalence of disease [14, 15]. The ratio of males to females in the study is about 1:1 which is similar to distribution of participants in another study done in 2019 [16].

The awareness campaign corrected the misconception that any kind of physical contact with a patient is responsible for transmitting the disease, as shown by the paired samples t-test. Many studies have detailed that exchanged of bodily fluids, for example blood, semen, vaginal secretions, as the only form of physical contact through which HBV and HCV can spread [17].

Another misconception that the campaign corrected was the myth that carriers of the disease can marry each other, as shown by the results of the study. A study conducted in China [18] explains the implications of marriage with regard to the disease. The study established that in addition to the financial burden experienced by the family, there is always the mystery of potential offspring being infected with the virus because mother to child transmission is indeed possible.

By the end of the activity, participants also realized that insects are responsible for spread of diseases, like dengue [13] and malaria, and not HBV or HCV. Similarly, administration of hepatitis B vaccine cannot protect against hepatitis C vaccine. The difference in genotypes of both viruses is one of the main reasons for exploring different vaccine options in studies [19].

There are a few limitations to the study. This is a survey and statistical analysis could not be done in detail as opposed to other study designs. The study population was focused on one community and so, has to include other communities as well. The format of the campaign was discussion based. Therefore, any participant who did not raise questions during the discussion, unfortunately, could not gain a lot from the activity.

Several attempts should be made to organize multiple activities on different levels of health care setup to ensure that the objective could be met. Additionally, instead of a discussion based format, it would be more appropriate if the format included more pictorial aids.

## Conclusions

The campaign provided a great degree of information regarding HBV and HCV but there is a dire need to expand such attempts so that the vulnerable population making up the majority of the nation can be protected.
